# Asymmetric Reproductive Isolation between Two Sympatric Annual Killifish with Extremely Short Lifespans

**DOI:** 10.1371/journal.pone.0022684

**Published:** 2011-08-05

**Authors:** Matej Polačik, Martin Reichard

**Affiliations:** Department of Fish Ecology, Academy of Sciences of the Czech Republic, Institute of Vertebrate Biology, Brno, Czech Republic; Auburn University, United States of America

## Abstract

**Background:**

Interspecific reproductive isolation is typically achieved by a combination of intrinsic and extrinsic barriers. Behavioural isolating barriers between sympatric, closely related species are often of primary importance and frequently aided by extrinsic factors causing spatial and temporal interspecific separation. Study systems with a severely limited role of extrinsic factors on reproductive isolation may provide valuable insights into how reproductive isolation between sympatric species is maintained. We used no-choice experimental set-up to study reproductive barriers between two closely related sympatric African killifish species, *Nothobranchius furzeri* and *Nothobranchius orthonotus*. These fish live in small temporary savannah pools and have complete spatial and temporal overlap in reproductive activities and share a similar ecology.

**Principal Findings:**

We found that the two species display largely incomplete and asymmetric reproductive isolation. Mating between *N. furzeri* males and *N. orthonotus* females was absent under standard experimental conditions and eggs were not viable when fish were forced to mate in a modified experimental setup. In contrast, male *N. orthonotus* indiscriminately mated with *N. furzeri* females, the eggs were viable, and offspring successfully hatched. Most spawnings, however, were achieved by male coercion and egg production and embryo survival were low. Behavioural asymmetry was likely facilitated by mating coercion from larger males of *N. orthonotus* and at relatively low cost to females. Interestingly, the direction of asymmetry was positively associated with asymmetry in post-mating reproductive barriers.

**Significance:**

We showed that, in fish species with a promiscuous mating system and multiple matings each day, selection for strong mate preferences was relaxed. This effect was likely due to the small proportion of resources allocated to each single mating and the high potential cost to females from mating refusal. We highlight and discuss the fact that males of rarer species may often coercively mate with females of a related, more abundant species.

## Introduction

The basis to defining a species is contingent on its delimitation as a unit whose reproduction is separated from that of other such units. This definition is valid for most species concepts. Insights into how species remain separated and identification of the barriers preventing interspecific gene flow, therefore, are considered as key issues in speciation and evolutionary studies [1].

Interspecific gene flow in sympatric species is usually restricted by a combination of intrinsic and extrinsic reproductive isolation barriers. Among intrinsic barriers, behavioural isolation is typically of primary importance [1]. Sympatric species discriminate between their own and other species and show a strong preference for mating with a conspecific partner. There is ample evidence that behavioural mate recognition is often based on colour and morphology, combined with specific male displays (e.g. cichlid fishes: [2], swordtail fishes: [3], lizards: [4], songbirds: [5], insects: [6]).

Behavioural reproductive isolation barriers are frequently reinforced by extrinsic factors, even in sympatric species with a high encounter rate. For example, coexisting species may be segregated at a relatively fine spatial scale [7], may have different habitat preferences [8], [9], or timing of reproduction [10]. The assumptions of a pivotal role of behavioural mate recognition, such as interspecific divergence in morphological traits and behavioural patterns are, however, not always met. At the same time, environmental constraints may prevent extrinsic factors from having any major effect on interspecific reproductive isolation. These exceptions may provide the most valuable insights into how reproductive isolation between sympatric species is maintained.

Small African annual killifish of the genus *Nothobranchius* (Nothobranchiidae, Cyprinodontiformes) are an example of a complex of around 50 species, with a frequent occurrence of 2–3 species in sympatry. There is relatively little interspecific divergence in terms of body size or shape [11] and, although male colour patterns are unusually variable across *Nothobranchius* spp., the difference may not be particularly distinct in some species living in sympatry (see below). Male spawning behaviour is simple and brief, males intercepting passing females and immediately attempting to spawn with them with no elaborate mating display. Though males are aggressive, they do not defend a territory [11], [12]. The fish inhabit small temporary savannah pools, often with a surface area of just a few square metres [13], [14]. Under these circumstances, any opportunity for spatial or habitat segregation is severely limited [13], as reflected, for example, by the diet of three sympatric species all preying on both benthic and limnetic prey and thus showing almost complete qualitative dietary overlap [14]. Further, the lifespan of *Nothobranchius* spp. is extremely short [15] as it is constrained by the longevity of the pools they inhabit. Savannah pools are restricted to the period of the rainy season (3–6 months), though many pools are filled with water for only 2–3 months (Polačik, Reichard, unpublished data). Within such a short period, the fish must hatch, mature and reproduce, and any delay in reproductive activities increases the risk of reproductive failure. Sympatric species, therefore, hatch at the same time and mature in 3–4 weeks [16], with their reproduction largely overlapping in time. After reaching sexual maturity they reproduce daily, with 20–40 single eggs laid each day in multiple spawning acts [12]. *Nothobranchius* are bottom substrate spawners with no specific requirements for spawning substrate [17], and hence their spawning also overlaps spatially.

The ecology and mating system of *Nothobranchius* spp. makes research on the mechanism of interspecific reproductive isolation barriers especially attractive. In *Nothobranchius* spp., the precision of behavioural reproductive isolation between sympatric species should be a result of the trade-off between choosiness that is sufficient to correctly discriminate heterospecific partners and strong selection against any delay in reproduction due to the brief reproductive opportunity in each reproductive event and limited time available for reproduction overall. Hence, *Nothobranchius* spp. are expected to have evolved a particularly precise mechanism of reproductive isolation in which conspecific partners are accepted extremely readily and heterospecifics are discriminated with high accuracy in order to cope with their unusually challenging environmental conditions, characterised by the lack of extrinsic factors enabling temporal and spatial species segregation.

**Figure 1:**
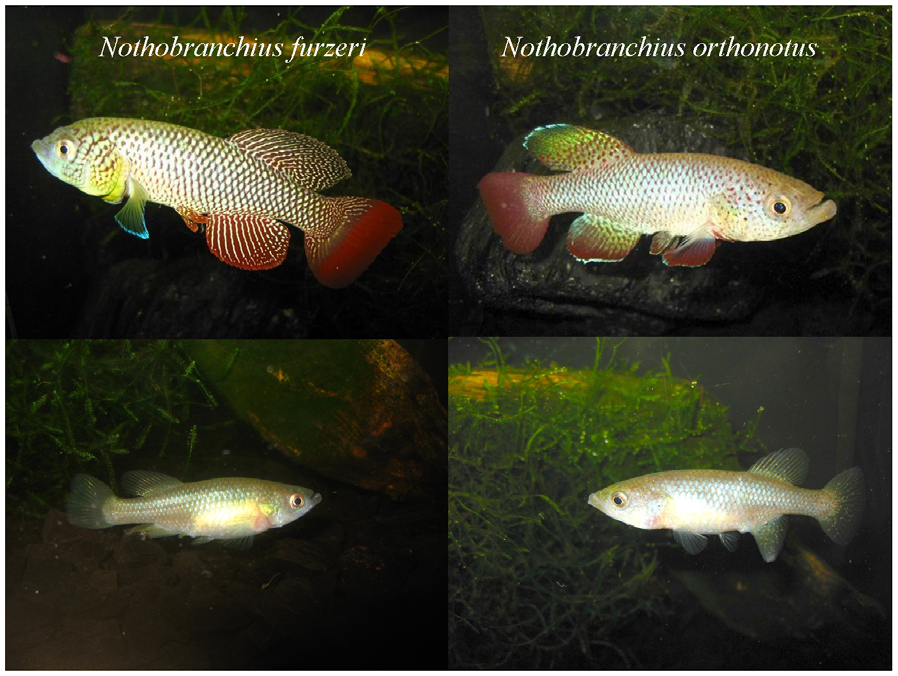
Photographs of sexually mature males (upper) and females (lower) of *Nothobranchius furzeri* (left) and *Nothobranchius orthonotus* (right). Photo by D. Kopeček.

In this study, we investigated reproductive isolation barriers between *Nothobranchius furzeri* Jubb and *Nothobranchius orthonotus* (Peters) under laboratory conditions. These two *Nothobranchius* species are closely related [11], [18] and share a large area of sympatry in southern Mozambique [13]. Both species are of similar size and colouration (Fig. 1). We predicted that stable coexistence of the two species is primarily based on behavioural reproductive isolation. We further predicted that fish restrained from reproduction for longer periods would be more prone to mate, and to mate more often with a heterospecific partner. In cases where interspecific spawning was possible, we predicted hybrid embryos to be unviable. These predictions are based on the apparently stable coexistence of the two species throughout their range [13]. We studied reproductive behaviour in a no-choice experimental setting and compared egg and embryo viability and hatching success between conspecific and heterospecific matings. Surprisingly, we found that behavioural reproductive isolation is largely incomplete, asymmetric, and that hybrid offspring are viable. We discuss our results in the context of male mate coercion.

## Materials and Methods

### Study species

Sexually mature individuals of both species typically reach 40–70 mm total length, though *N. orthonotus* tend to be marginally larger [16]. Males have similar colouration, the body being iridescent pale blue-green with red markings [11] and anal and caudal fins dominated by red colouration (Fig. 1). *N. orthonotus* differs in having a white marginal band on the dorsal and anal fins, whereas *N. furzeri* is characterised by a white marginal band on the pectoral fins. The dorsal profile of the head is slightly concave in *N. orthonotus* and moderately convex in *N. furzeri*. This trait is useful for distinguishing females, which are otherwise the same colour, i.e. brown with transparent fins (Fig. 1).

The *N. furzeri* and *N. orthonotus* used in the experiment originated from sympatric populations collected from the Limpopo river basin in southern Mozambique (collection code MZCS08-122, GPS coordinates S 24°18.207′E33°02.779′) in February 2008. Five males and eight females of *N. furzeri*, and six males and eight females of *N. orthonotus* were used to found the captive populations. The experimental fish were the F2 generation (imposed laboratory breeding protocol to maximise outbreeding) and were of exactly the same age. Hatched offspring of the two species were housed separately in a series of 12 litre tanks (to avoid interspecific competition and predation as *N. orthonotus* is larger at hatching). The position of each tank, however, ensured that fish of the two species had mutual visual contact. The diet initially consisted of *Artemia* nauplii. After 15 days, the diet was changed to frozen *Chironomus* larvae and the fish were transferred into four 100 litre (57×50×35 cm) tanks, with interspecific visual contact retained. During this phase, two of the four tanks contained 10 pairs of *N. furzeri* and the other two contained 10 pairs of *N. orthonotus*. Ten days before the start of the experiment, both species were mixed and each of the four tanks contained five pairs of *N. furzeri* and five pairs of *N. orthonotus*. The experiment was performed when the fish were six weeks old, i.e. 2 weeks after all fish reached sexual maturity, and consisted of behavioural observations and estimates of egg survival and hatching success.

### Spawning behaviour

Heterospecific and conspecific (control) pairs were allowed to spawn following a period of short (24 hours) and long (72 hours) restraint from reproduction and their spawning behaviour recorded. Both the short and long restraint periods were achieved by isolating females from males. The females were isolated in order to standardise female fecundity [19] and enable comparison of egg production (see below). The two periods were used to test whether duration of reproductive restraint affected the predisposition of females to spawn with heterospecific (and conspecific) males.

Spawning behaviour was recorded for 80 pairs. Ten pairs of each of the four combinations possible (male *N. furzeri*×female *N. furzeri* - henceforth denoted as FF, male *N. orthonotus*×female *N. orthonotus* - OO, male *N. furzeri*×female *N. orthonotus* - FO, male *N. orthonotus*×female *N. furzeri* - OF) were tested after the 24 h female isolation. After testing, the females were isolated from males again for 72 hours then tested again in the same (conspecific or heterospecific) treatment. We were unable to use a new set of individuals or keep records of individual fish between the first and second isolation period due to logistic constraints and interference with some aspects of the experimental design. Each male and female was tested twice, therefore, though with a different partner. In order to avoid pseudo-replication, we analysed data obtained for treatments with isolation of 24 and 72 hours separately. Mean (± s.e.) standard length (measured from the tip of snout to the end of the caudal peduncle) was 37.6±4.5 mm in male *N. orthonotus*, 35.9±3.6 in male *N. furzeri*, 31.8±2.3 mm in female *N. orthonotus* and 28.3±2.1 mm in female *N. furzeri*.

The setup for the observation of reproductive behaviour consisted of a 100 litre (57×50×35 cm) tank divided into two halves by a perforated glass slide. The slide, enabling both visual and olfactory communication, formed a barrier between the experimental males and females during the initial settling period. After one hour of limited contact, the male was gently captured using a hand net and placed into the female compartment. The female compartment contained a shallow dish (80 mm in diameter, depth 15 mm) filled with fine black sand, which acted as a spawning substrate. Recording of fish behaviour started when the fish began to interact or 10 min after the male had been introduced, whichever happened sooner. Four behavioural categories were recorded that included the entire range of fish behaviour during the experiment. Behavioural categories were: (1) “no interaction”, (2) “chasing”, (3) “forced spawning” and (4) “consent spawning” (Table 1). Fish behaviour was recorded every minute within the observation period of 20 min. At the end of the experimental trial, eggs spawned in the dish were counted in order to provide an additional measure of the spawning success of the pair.

**Table 1:** Description of four behavioural categories recorded during experimental observations of conspecific and heterospecific pairs.

Behavioural category	Definition
No interaction	Neither fish showed any interest in the tank mate.
Chasing	The male quickly swam after the female in the water column without succeeding in forcing her to the bottom of the tank.
Forced spawning	The male attempted to spawn with the female on the bottom of the tank by folding his dorsal fin around her, but the female resisted the male's clasp and tried to escape. This category of behaviour was always performed outside the spawning dish.
Consent spawning	A typical repeated spawning event, always taking place in the spawning dish, presumably with egg deposition.

### Egg survival and hatching success

After counting, all eggs spawned into the spawning dish were incubated in order to obtain data on egg survival rate and hatching success. The eggs were placed on a substrate composed of damp peat and stored in covered Petri dishes in a dark, temperature-controlled incubation box (Q-cell 140/50, Poll Lab Ltd.) at 25°C. Eggs from each replicate were stored in a separate dish and all dishes were monitored once daily during the first five days and every third day throughout the rest of the incubation period. Any dead eggs were removed to avoid fungal infections affecting the surviving eggs. Egg survival was estimated as the proportion of eggs produced that developed successfully to the pre-hatching stage, easily determined by the appearance of gold-pigmented eyes in the embryo. At this developmental stage, the embryo is ready to hatch if the egg is placed into water [20]. Hatching success was estimated as the proportion of eggs scored as “survived until the pre-hatching state” that successfully departed from the eggshell and started to swim after placing in water.

Additional batches of eggs were needed to test survival and hatching success in heterospecific pairings as too few eggs were laid in many experimental replicates. To produce additional batches, a pair of fish (20 pairs OF, 20 pairs FO in total) was placed for one hour into a small 2 litre plastic container (15×10×12 cm) with a black sand spawning substrate covering the entire floor of the container. No behavioural observations were performed during the additional matings, other than for monitoring fish welfare.

### Data analysis

Differences in fish behaviour and the parameters of reproductive success were analysed using General Linear Models (GLM, normally distributed data) and Generalised Linear Models (GLLM, data with non-normal distribution). Parental combination (FF, OO, FO, OF) was the fixed factor, with female body size entered as a covariate in the analysis of egg production. A quasi-poisson distribution was used for count data as many data points were equal to zero. Models were first tested using a Poisson distribution, and potential overdispersion then tested using the ratio of residual deviance to residual degrees of freedom (parameter theta). In all cases, theta departed from 1 and a quasi-poisson distribution was used to account for overdispersion in significance testing. Note that the use of traditional (but less powerful) non-parametric methods (e.g. Kruskall-Wallis test, Median test) provided entirely concordant outcomes. In linear models on proportional data (hatching success), data were arcsine square-root transformed prior to analysis to normalise residuals.

For survival rate and hatching success, no distinction was made between eggs produced after differential sex separation. The measure of hatching success was independent of egg survival since it was calculated as a proportion of the eggs that hatched from those surviving to the pre-hatching stage. Only batches with at least two eggs were included in survival and hatching analyses. Concordant results for survival rates were also obtained when only batches >3 or >5 eggs were included. For hatching rate, increasing the threshold value for batch size decreased the number of eligible observations; although the results remained unchanged, their power decreased considerably. The final sample size for egg survival rate analysis was 15, 12, 31 and 19 batches of eggs for FF, FO, OF and OO combinations, respectively. For hatching rate, 7, 6 and 7 batches for FF, OF and OO combinations, respectively, were included in the analysis. Independence of measures for survival and hatching were tested using Spearman correlation between the survival rates of particular batches of eggs and their hatching success. Statistical analyses were performed using R package 2.9.1 (R Development Core Team, 2009).

## Results

**Figure 2:**
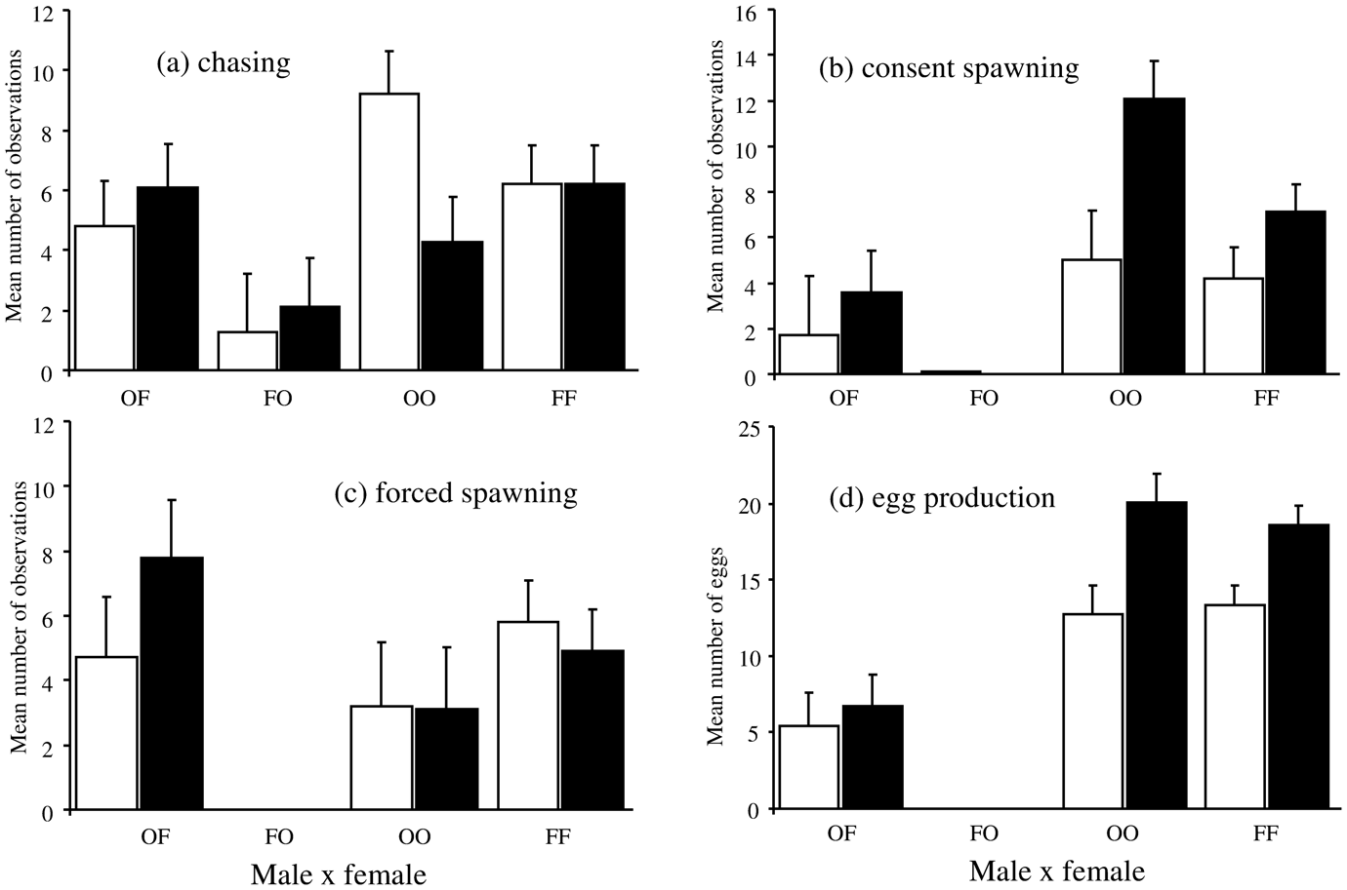
Male behaviour and egg production recorded during experimental trials (mean and one standard error). The incidence of chasing (a), consent spawning (b), forced spawning (c), and the mean number of eggs recovered (d) are shown for each parental combination (FF = male *N. furzeri*×female *N. furzeri*, OO = male *N. orthonotus*×female *N. orthonotus*, FO = male *N. furzeri*×female *N. orthonotus*, OF = male *N. orthonotus*×female *N. furzeri*) and period of isolation (24 h: white bars, 72 h: black bars).

The rate of male chasing was affected by parental combination when females were separated for 24 hours (GLLM with quasi-poisson error structure, F_3,36_ = 5.16, P = 0.005), but no significant effect was observed after 72 hours of separation (F_3,36_ = 2.04, P = 0.126), probably due to increased interest in *N. orthonotus* females by *N. furzeri* males after separation (Fig. 2a).

The number of consent spawnings was highest in conspecific treatments (GLLM with quasi-poisson error structure, 24 h: F_3,36_ = 5.21, P = 0.004; 72 h: F_3,36_ = 17.52, P<0.001) and more consent spawnings were observed after a 72 h separation compared to a 24 h separation period, except for *N. furzeri* males paired with *N. orthonotus* females where only a single consent spawning was observed (Fig. 2b).

The number of forced spawnings also differed among treatments, being absent in heterospecific combination with *N. furzeri* males but present in all other combinations at a comparable rate (GLLM with quasi-poisson error structure, 24 h: F_3,36_ = 7.48, P = 0.001; 72 h: F_3,36_ = 12.10, P<0.001; Fig. 2c).

During behavioural observation trials, females laid more eggs with conspecific partners (GLLM with quasi-poisson error structure, 24 h: F_3,36_ = 6.79, P = 0.001; 72 h: F_3,36_ = 10.37, P<0.001). The number of eggs laid was not affected by female body size (24 h: F_1,35_ = 3.64, P = 0.065; 72 h: F_1,35_ = 0.37, P = 0.545). Heterospecific combinations with *N. orthonotus* males produced an intermediate number of eggs (about one third of those recorded for conspecific combinations), while no eggs at all were collected from heterospecific treatments with *N. furzeri* males. Increased time of separation increased the number of eggs produced, and this trend was general across all treatments where eggs were laid (Fig. 2d).

**Figure 3:**
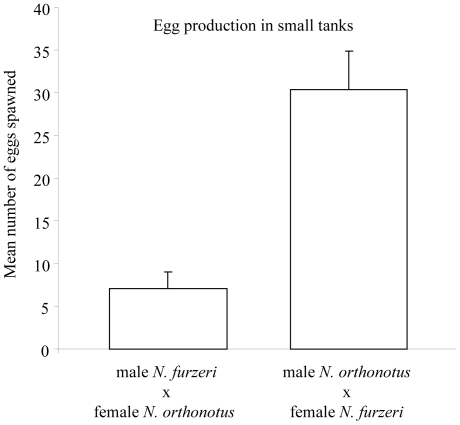
Mean (+ standard error) number of eggs spawned by heterospecific pairs in small 2 litre tanks.

Contrary to the pairings in larger tanks, both heterospecific combinations in small containers spawned eggs. The mean number of eggs produced by the male *N. furzeri*×female *N. orthonotus* combination, however, remained much lower than that by the male *N. orthonotus*×female *N. furzeri* combination (GLLM with quasi-poisson error structure, F_1,38_ = 312.82, P = 0.001; P<0.001, Fig. 3).

**Figure 4:**
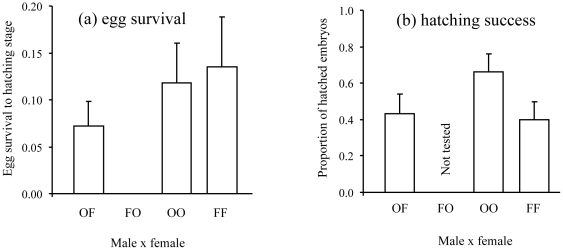
Mean (+ standard error) egg survival to hatching stage (a), and proportion of hatched embryos (b), for each parental combination (FF = male *N. furzeri*×female *N. furzeri*, OO = male *N. orthonotus*×female *N. orthonotus*, FO = male *N. furzeri*×female *N. orthonotus*, OF = male *N. orthonotus*×female *N. furzeri*). The proportion of hatched embryos was estimated from embryos surviving to the hatching stage, with the two measures independent.

Egg survival differed among treatments (GLLM with quasi-poisson error structure, F_3,73_ = 3.50, P = 0.020), with greatest survival in conspecific combinations and zero survival in the *N. furzeri* male with *N. orthonotus* female combination (Figure 4a). There was no difference in hatching success among treatments for eggs that survived until the pre-hatching stage (GLM, normal distribution, arcsine square-root transformed, F_2,17_ = 1.79, P = 0.197, Fig. 4b). The *N. furzeri* male×*N. orthonotus* female combination was omitted from this analysis as no embryos survived to the pre-hatching stage. Hatching success of specific batches was not correlated with their survival during embryonic development (Spearman correlation, *r*_S_ = 0.162, N = 33, P = 0.367).

## Discussion

We found that two sympatric killifish species with a similar ecology and complete spatial and temporal overlap in reproductive activities displayed a largely incomplete and asymmetric reproductive isolation. Asymmetry in behavioural reproductive isolation was associated with asymmetry in post-mating reproductive barriers. Spawnings between *N. furzeri* males and *N. orthonotus* females were virtually absent under standard conditions and eggs were not viable when fish were forced to mate in a modified experimental setup. In contrast, male *N. orthonotus* spawned with *N. furzeri* females, the eggs were viable, and offspring successfully hatched. Most spawnings, however, were achieved by male coercion in no-choice situation and egg production and embryo survival were lower than in conspecific mating. Isolation of experimental fish from the opposite sex for a longer period affected female willingness to mate. The trend, however, was concordant among all parental combinations (except for the combination with no mating) rather than only promoting more interspecific matings.

Incomplete reproductive isolation barriers are often reported among closely related sympatric species [1], but are typically coupled with isolation by extrinsic factors such as fine-scale spatial isolation of breeding sites [7], [8], [21], or differential timing of reproduction (reviewed in [22]). The experimental results contradict our prediction that, in the absence of extrinsic barriers to interspecific mating and a high encounter rate between two closely related species, intrinsic barriers should be particularly efficient and primarily manifested as behavioural, pre-mating barriers. We believe that the incompleteness of the reproductive barriers stems from the specific life history and mating system of our study taxon, and from differential costs associated with interspecific mating for males and females.

All *Nothobranchius* species have a promiscuous mating system with repeated spawning every day and no parental care. The fish hatch as soon as their pool fills with water at the onset of the rainy season; whereupon juveniles grow rapidly and become sexually mature within 3–4 weeks. Both species start to reproduce immediately after achieving sexual maturity, with individual females producing 20–40 eggs each day, distributed among several mating events and, typically, among several males [12], (M. Polačik, M. Reichard unpublished data). Males do not defend a territory and attempt to mate with any passing female [12]. As eggs are laid individually, investment in a single mating is relatively low for both sexes, while selection against postponing reproduction is strong due to the unpredictability of habitat duration. We believe that the relatively low cost (in terms of time and energy needed to produce gametes) of each spawning act may relax selection on the strength of mate choice as an occasional heterospecific spawning does not constitute a significant cost compared to a missed opportunity for conspecific spawning. This scenario, however, inherently presupposes that hybrids, despite being partly viable, are not fertile and the species thereby remain reproductively isolated. Indirect evidence of hybrid sterility between various *Nothobranchius* species can be found in the killifish hobbyist literature on *Nothobranchius* breeding; these fish are popular with aquarists who maintain many colour-aberrant strains. Despite numerous attempts, none of the present colour-aberrant strains produced by breeders come from interspecific crossings as these invariably fail to produce lineages persistent beyond several (typically F2) generations [23], [24]. In the present study, we were primarily interested in behavioural barriers between species and hybrid sterility was not rigorously tested. Hybrid offspring between male *N. orthonotus* and female *N. furzeri* did produce eggs, but they invariably failed to develop to the pre-hatching stage (a total of more than 100 eggs). No backcrosses were tested.

Reproductive isolation in both pre- and post-zygotic barriers between *N. furzeri* and *N. orthonotus* was asymmetric. Asymmetries in reproductive isolation are frequently reported when interspecific hybridisation occurs [25], [26], [27], typically arising from genetic constraints or asymmetric costs for the particular species or sex [25], [28]. In our study system, a combination of asymmetry in sex-specific reproductive costs and genetic incompatibility are both plausible explanations. All known examples of sex determination in *Nothobranchius* are genetic, but different species possess different sex determination systems [29], [30]. In *N. furzeri*, males are heterogametic sex with XY/XX system with sex-determining region located on autosomal chromosome [30], though multiple sex chromosome system was identified in *N. guentheri* [29]. Nothing is known on sex-determining system in *N. orthonotus*, but sex-specific hybrid inviability may result from interspecific differences in sex-determination system or position of sex-determining regions. Another reason for genetic incompatibility may relate to marked cytogenetic differences between *N. furzeri* and *N. orthonotus*, with *N. furzeri* having pericentromeric region unusually enriched with satellite-sequences, absent in *N. orthonotus* [31].

We hypothesise that asymmetry is generated by male coercion. Male *N. orthonotus* attempted to mate with heterospecific females with similar vigour as with conspecifics (Fig. 2a), whereas male *N. furzeri* showed no behavioural response to *N. orthonotus* females. Most spawnings between male *N. orthonotus* and female *N. furzeri* were, however, achieved by male coercion (Fig. 2b, c). Notably, *N. orthonotus* is generally very aggressive and males may often injure females during mating [11]. As in most other organisms, the cost of reproduction in *Nothobranchius* is lower for males compared to females. First, sperm production is relatively less expensive than egg production. Second, *Nothobranchius* males invest little time in female attraction and lack elaborate displays that are energetically expensive or increase the risk of predation [12]. Under these circumstances, postponing any mating attempt would be maladaptive, especially when the costs of a mistaken refusal of a conspecific female are likely to be higher than those of mistaken acceptance of a heterospecific female [28] given that habitat longevity is short, e.g. [32]. Hence, we hypothesise that male *N. orthonotus* vigorously attempt to mate even with heterospecific females due to the negligible costs associated with a heterospecific spawning. Notably, we have also observed the same male behaviour in the field, where male *N. orthonotus* intercepted heterospecific females and forced them to spawn (M. Polačik, M. Reichard, personal observation), discounting the possibility that the observed failure in behavioural reproductive barrier was solely an experimental artefact.

In contrast, male *N. furzeri* showed no interest in heterospecific females in the large experimental tanks. *Nothobranchius furzeri* are less aggressive than *N. orthonotus* in general, including during mating. It is possible that artificial spatial confinement prevented otherwise efficient behavioural barriers to mating as *N. furzeri* males did spawn with *N. orthonotus* females in small tanks, perhaps due to the female's inability to move beyond the critical distance that would discourage the male from repeated mating attempts. Another possible explanation of the lack of interest shown by *N. furzeri* males in heterospecific females may be linked to genetic incompatibility causing complete inviability of the hybrid progeny (Fig. 4b) and consequent reinforcement of behavioural discrimination. This implies, however, that mating between *N. orthonotus* males and *N. furzeri* females results in some introgression (non-zero reproductive success of *N. orthonotus* males mating with *N. furzeri* females). While this has been reported in spadefoot toads [33], the support in our study system is weak [34].

Another intriguing point arises from the direction of reproductive isolation asymmetry. The lack of discrimination of *N. orthonotus* males is difficult to explain in terms of relative species abundance in the wild. In most habitats where *N. orthonotus* and *N. furzeri* co-occur, *N. orthonotus* is consistently less abundant [14], with *N. orthonotus* abundance being 5–30% that of *N. furzeri*, including the site of origin of our study populations (15%, M. Reichard and M. Polačik, unpublished data). This difference in relative abundance means that most females encountered by *N. orthonotus* males (which mated indiscriminately) in the wild are likely to be *N. furzeri* females. It is possible that some factors that cannot be measured in the laboratory may cause latency of *N. orthonotus* males to mate under natural conditions (e.g. environmental water turbidity) and could affect the natural rate of heterospecific mating or mating attempts. However, there are reports of the same pattern of asymmetry in male-initiated mating with heterospecific partners in other pairs of co-occurring, closely related species. For example, Hettyey et al. [28] reported the same asymmetry pattern in male-initiated mating with heterospecific partners; males of the rarer species (frog *Rana temporaria*) were indiscriminate, readily pairing with heterospecific females and hence were more likely to pair with heterospecific (*Rana dalmatina*) females. Males of the rare Grevy's zebra (*Equus grevyi*) mate with females of the smaller, abundant plains zebra (*Equus burchelli*). Hybrid offspring are viable and at least the female F1 hybrids are fertile [35]. It is likely that sexual coercion by Grevy's zebra males stems from the low abundance of conspecific females and the larger size of Grevy's zebra males [35], a situation comparable to our study system.

Costs associated with resistance to mating in female *Nothobranchius* may exceed the costs of heterospecific mating. Hence females, despite being able to discriminate against heterospecific males, may opt to accept any partner in order to avoid injuries resulting from male harassment. High costs of female refusal to mate are reported for many species, including cases of fatal injuries (reviewed in [36]). We frequently noticed (including field observations) overt male aggression against females that resisted male mating attempts, an observation also reported by [11]. Female *N. furzeri* did mate with *N. orthonotus* males, despite an apparent ability to discriminate, as manifested through the significantly elevated proportion of forced rather than consent spawnings (Fig. 2b, c) and the decreased number of spawned eggs (25–29%; Fig. 2d) compared to conspecific pairings. We believe that the occasional willingness of *N. furzeri* females to accept the heterospecific male may be adaptive, despite some reproduction costs, as females thereby decrease the risk of physical injury by the male. Although space limitation under experimental conditions may have additionally restricted the female's ability to avoid males, similar spatial limitation also frequently arises in the small pools in which these fish are frequently found in the wild. Moreover, the character of the mating act itself (with the female being pushed by the male towards the bottom from above) further limits female control of spawning. In conclusion, a small number of eggs spawned by a female during a forced mating may increase the likelihood of being released from harassment by the male, as reported in frogs [28].

In summary, our study showed that pre-mating reproductive barriers between two sympatric annual killifish species are incomplete and asymmetric. The behavioural asymmetry is probably facilitated by male mating coercion and associated costs to females of resistance to males. Larger and more aggressive *N. orthonotus* males may be able to enforce heterospecific mating. Given the *Nothobranchius* reproductive strategy, female costs associated with mating refusal (injuries from male harassment) may be higher than costs associated with heterospecific mating (loss of eggs). The direction of pre-mating asymmetry is consistent with asymmetry in post-mating barriers, with male *N. orthonotus* able to produce viable F1 progeny with *N. furzeri* females, while the combination of *N. furzeri* males with *N. orthonotus* females produced embryos that died before developing to the hatching stage. Future studies should test hybrid fertility and viability beyond the F1 generation and use microsatellite markers to investigate potential introgression in the wild.
